# Supplementary material for: Pentoxifylline decreases post-operative intra-abdominal adhesion formation in an animal model
Source: PeerJ. 2018 Aug 24;6:e5434. doi: 10.7717/peerj.5434 (PMC6110259; doi:10.7717/peerj.5434)
Supplement: Supplemental Information 1 [file peerj-06-5434-s001.pdf]

Related to Fig. 2A

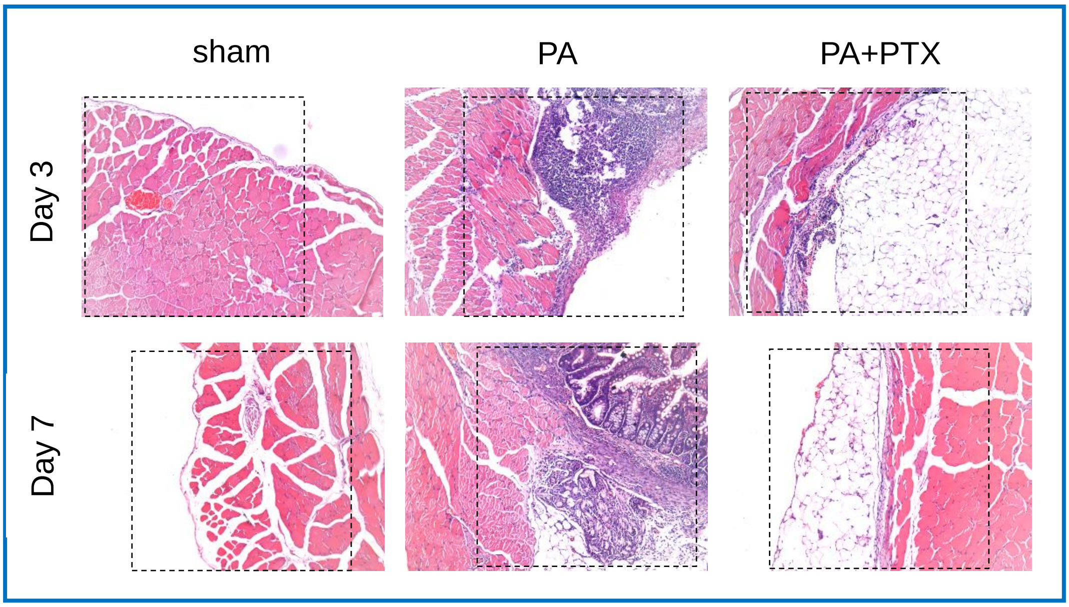

Original magnification ×200

Related to Fig. 2B

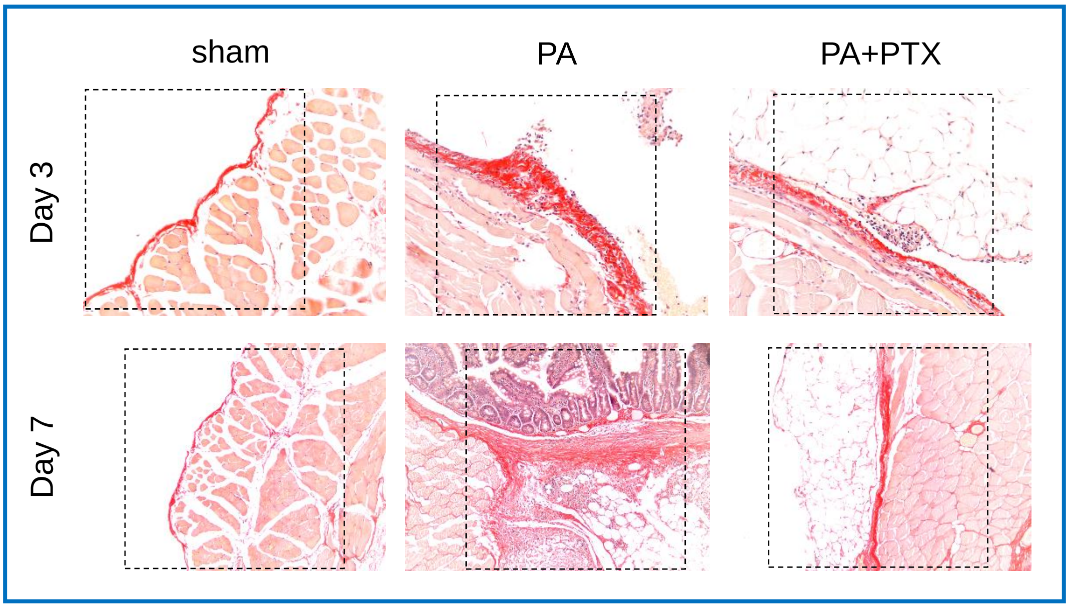

Original magnification ×400 (upper figure)
Original magnification ×200 (lower figure)

Related to Fig. 4A

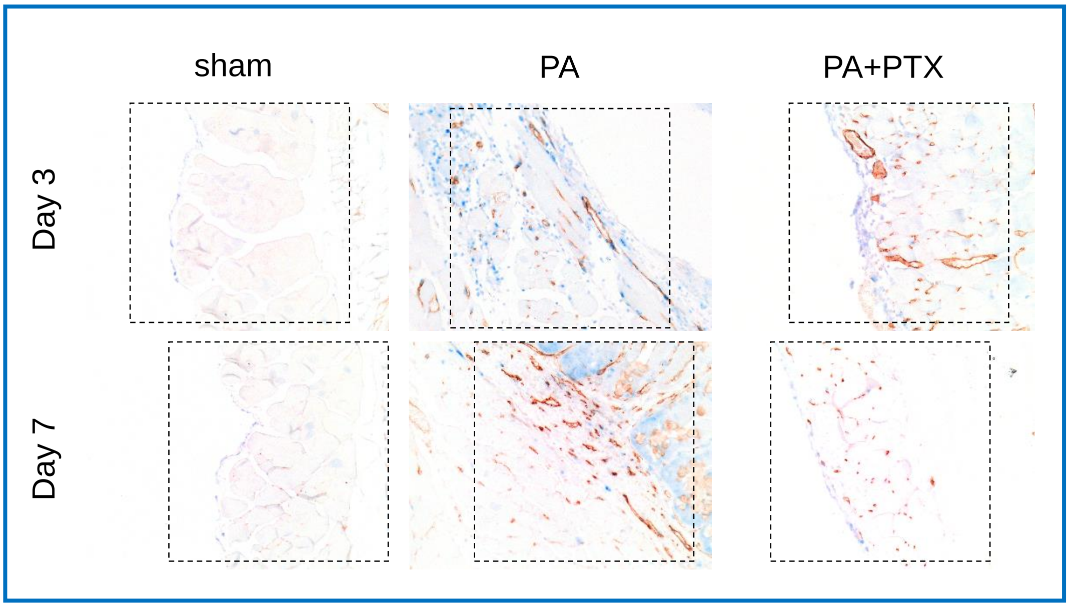

Original magnification ×200

Related to Fig. 5A

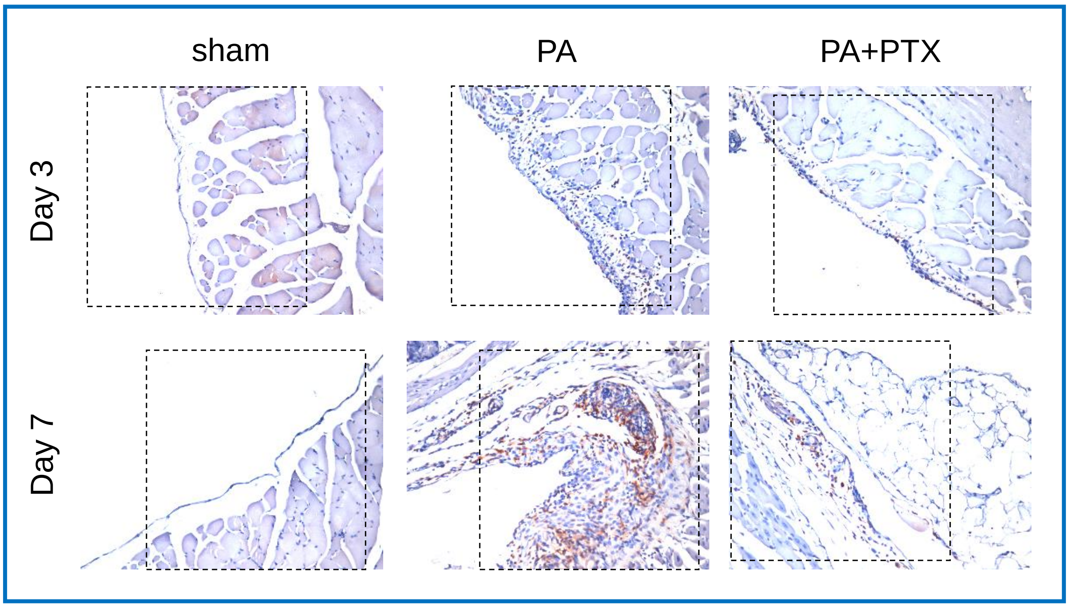

Original magnification ×200

Related to Fig. 6A

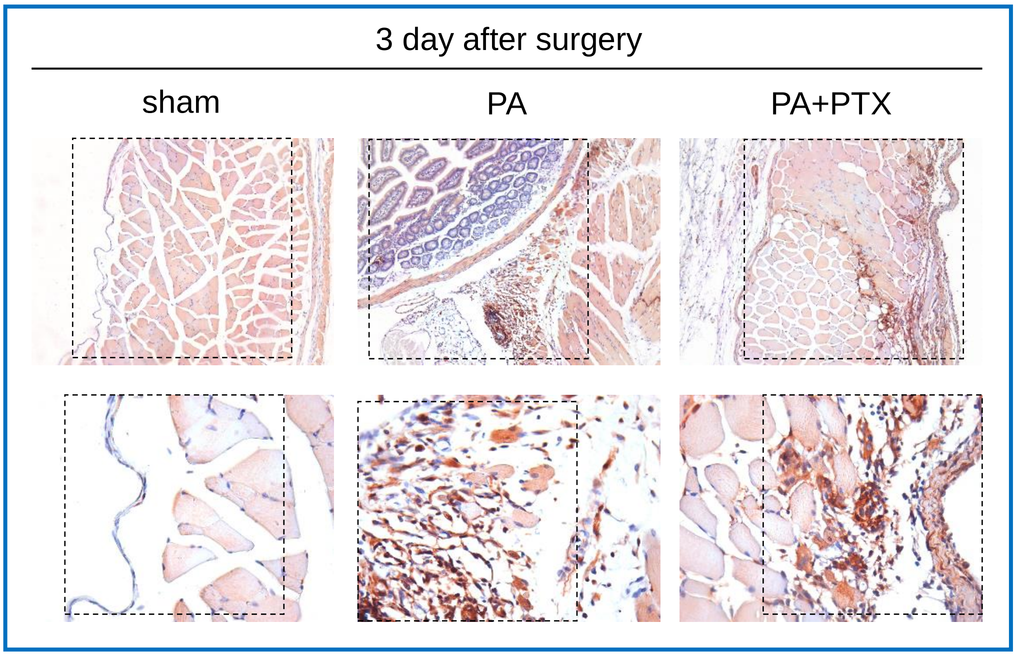

Original magnification ×100 (upper figure)

Original magnification ×400 (lower figure)

Related to Fig. 6B

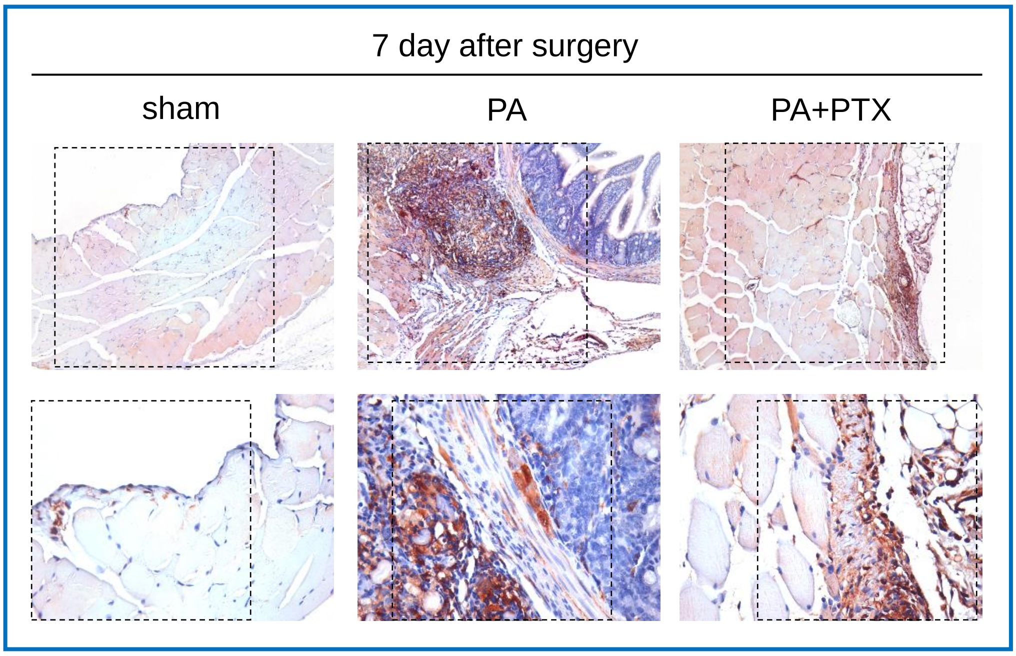

Original magnification ×100 (upper figure)

Original magnification ×400 (lower figure)

Related to Fig. 7A

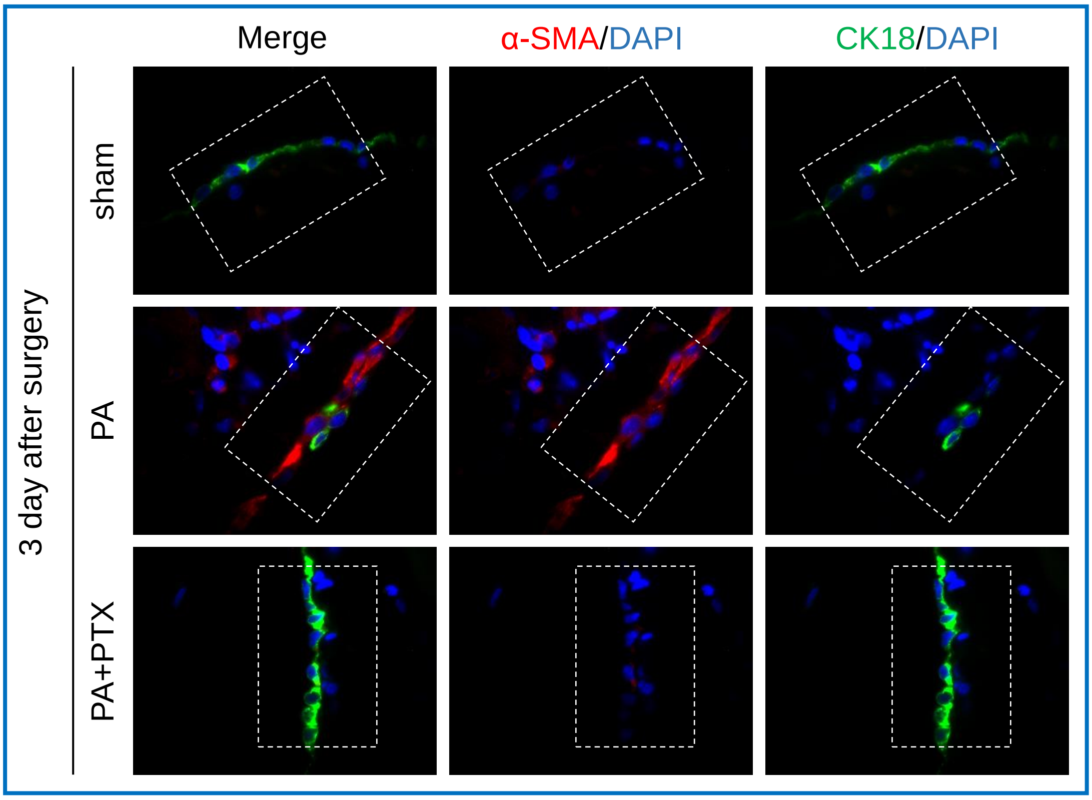

Original magnification ×1000

Related to Fig. 7B

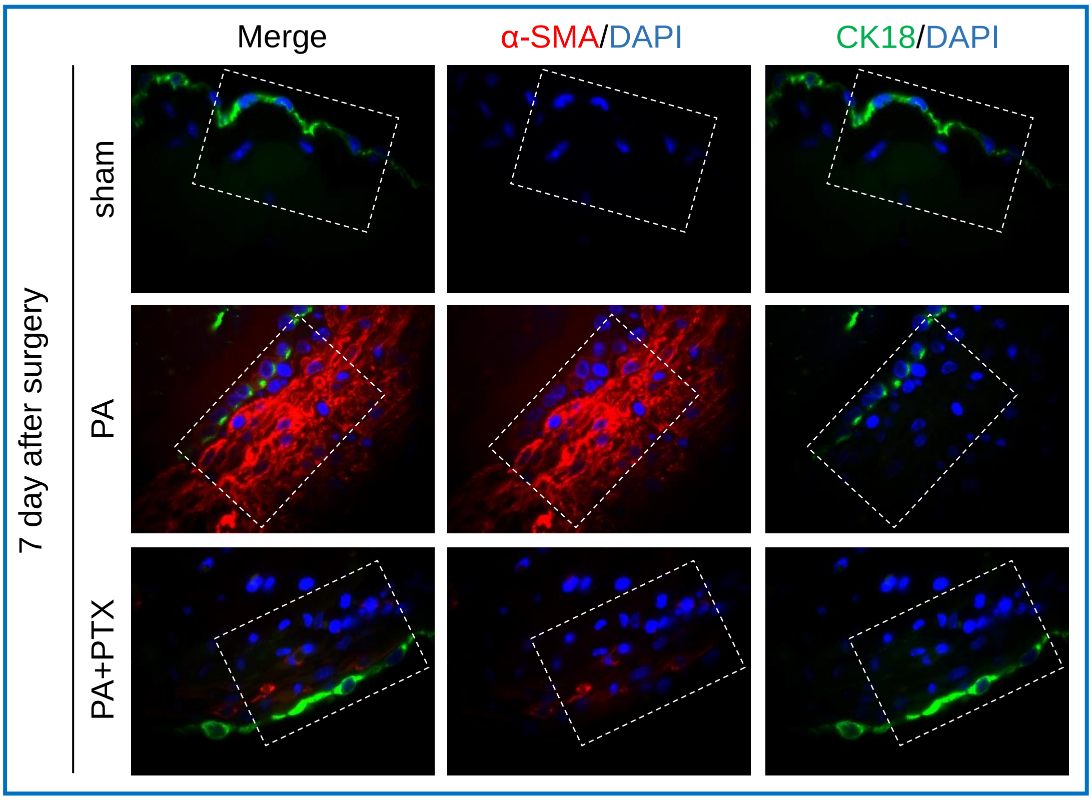

Original magnification ×1000
