# Supplementary material for: Pentoxifylline decreases post-operative intra-abdominal adhesion formation in an animal model
Source: PeerJ. 2018 Aug 24;6:e5434. doi: 10.7717/peerj.5434 (PMC6110259; doi:10.7717/peerj.5434)
Supplement: Supplemental Information 2 — Results from statistical analyses. [file peerj-06-5434-s002.docx]

Supplemental Table S1. Statistical comparison of intra-adhesion score between the groups. Related to Fig. 1.

| Groups | | *P* value | Summary |
| --- | --- | --- | --- |
| Day 3 | *sham* vs. *PA* | <0.0001 | *** |
|  | *PA* vs. *PA+PTX* | 0.0165 | * |
|  | *sham* vs. *PA+PTX* | 0.0025 | ** |
| Day 7 | *sham* vs. *PA* | <0.0001 | *** |
|  | *PA* vs. *PA+PTX* | 0.0267 | * |
|  | *sham* vs. *PA+PTX* | 0.0027 | ** |

Supplemental Table S2. Statistical comparison of tPA between the groups. Related to Fig. 3.

| Groups | | *P* value | Summary |
| --- | --- | --- | --- |
| Day 3 | *sham* vs. *PA* | <0.0001 | *** |
|  | *PA* vs. *PA+PTX* | 0.1921 | ns. |
|  | *sham* vs. *PA+PTX* | <0.0001 | *** |
| Day 7 | *sham* vs. *PA* | 0.0003 | *** |
|  | *PA* vs. *PA+PTX* | 0.0002 | *** |
|  | *sham* vs. *PA+PTX* | <0.0001 | *** |

Supplemental Table S3. Statistical comparison of ki67; CD31 staining between the groups. Related to Fig. 4B.

| Groups | | *P* value | Summary |
| --- | --- | --- | --- |
| Day 3 | *sham* vs. *PA* | <0.0001 | *** |
|  | *PA* vs. *PA+PTX* | <0.0001 | *** |
|  | *sham* vs. *PA+PTX* | 0.3751 | ns. |
| Day 7 | *sham* vs. *PA* | <0.0001 | *** |
|  | *PA* vs. *PA+PTX* | <0.0001 | *** |
|  | *sham* vs. *PA+PTX* | 0.9505 | ns. |

Supplemental Table S4. Statistical comparison of CD31^+^ staining between the groups. Related to Fig. 4C.

| Groups | | *P* value | Summary |
| --- | --- | --- | --- |
| Day 3 | *sham* vs. *PA* | 0.1636 | ns. |
|  | *PA* vs. *PA+PTX* | 0.3765 | ns. |
|  | *sham* vs. *PA+PTX* | 0.0700 | ns. |
| Day 7 | *sham* vs. *PA* | <0.0001 | *** |
|  | *PA* vs. *PA+PTX* | <0.0001 | *** |
|  | *sham* vs. *PA+PTX* | 0.9505 | ns. |

Supplemental Table S5. Statistical comparison of F4/80^+^ staining between the groups. Related to Fig. 5B.

| Groups | | *P* value | Summary |
| --- | --- | --- | --- |
| Day 3 | *sham* vs. *PA* | 0.0054 | ** |
|  | *PA* vs. *PA+PTX* | 0.0619 | ns. |
|  | *sham* vs. *PA+PTX* | 0.0619 | ns. |
| Day 7 | *sham* vs. *PA* | 0.0015 | ** |
|  | *PA* vs. *PA+PTX* | 0.0034 | ** |
|  | *sham* vs. *PA+PTX* | 0.0711 | ns. |

Supplemental Table S6. Statistical comparison of S100A4^+^ staining between the groups. Related to Fig. 6B.

| Groups | | *P* value | Summary |
| --- | --- | --- | --- |
| Day 3 | *sham* vs. *PA* | 0.0515 | ns. |
|  | *PA* vs. *PA+PTX* | 0.0177 | * |
|  | *sham* vs. *PA+PTX* | 0.7320 | ns. |
| Day 7 | *sham* vs. *PA* | 0.0670 | ns. |
|  | *PA* vs. *PA+PTX* | 0.0411 | * |
|  | *sham* vs. *PA+PTX* | 0.4722 | ns. |

Supplemental Table S7. Statistical comparison of α-SMA^+^ staining between the groups. Related to Fig. 7C.

| Groups | | *P* value | Summary |
| --- | --- | --- | --- |
| Day 3 | *sham* vs. *PA* | 0.0280 | * |
|  | *PA* vs. *PA+PTX* | 0.0337 | * |
|  | *sham* vs. *PA+PTX* | 0.4509 | ns. |
| Day 7 | *sham* vs. *PA* | 0.0159 | * |
|  | *PA* vs. *PA+PTX* | 0.0017 | ** |
|  | *sham* vs. *PA+PTX* | 0.4164 | ns. |
